# Deep learning‐based convolutional neural network for intramodality brain MRI synthesis

**DOI:** 10.1002/acm2.13530

**Published:** 2022-01-19

**Authors:** Alexander F. I. Osman, Nissren M. Tamam

**Affiliations:** ^1^ Department of Medical Physics Al‐Neelain University Khartoum 11121 Sudan; ^2^ Department of Physics College of Science Princess Nourah bint Abdulrahman University P. O. Box 84428 Riyadh 11671 Saudi Arabia

**Keywords:** brain cancer, convolutional neural network, deep learning, magnetic resonance imaging (MRI), medical imaging synthesis

## Abstract

**Purpose:**

The existence of multicontrast magnetic resonance (MR) images increases the level of clinical information available for the diagnosis and treatment of brain cancer patients. However, acquiring the complete set of multicontrast MR images is not always practically feasible. In this study, we developed a state‐of‐the‐art deep learning convolutional neural network (CNN) for image‐to‐image translation across three standards MRI contrasts for the brain.

**Methods:**

BRATS’2018 MRI dataset of 477 patients clinically diagnosed with glioma brain cancer was used in this study, with each patient having T1‐weighted (T1), T2‐weighted (T2), and FLAIR contrasts. It was randomly split into 64%, 16%, and 20% as training, validation, and test set, respectively. We developed a U‐Net model to learn the nonlinear mapping of a source image contrast to a target image contrast across three MRI contrasts. The model was trained and validated with 2D paired MR images using a mean‐squared error (MSE) cost function, Adam optimizer with 0.001 learning rate, and 120 epochs with a batch size of 32. The generated synthetic‐MR images were evaluated against the ground‐truth images by computing the MSE, mean absolute error (MAE), peak signal‐to‐noise ratio (PSNR), and structural similarity index (SSIM).

**Results:**

The generated synthetic‐MR images with our model were nearly indistinguishable from the real images on the testing dataset for all translations, except synthetic FLAIR images had slightly lower quality and exhibited loss of details. The range of average PSNR, MSE, MAE, and SSIM values over the six translations were 29.44–33.25 dB, 0.0005–0.0012, 0.0086–0.0149, and 0.932–0.946, respectively. Our results were as good as the best‐reported results by other deep learning models on BRATS datasets.

**Conclusions:**

Our U‐Net model exhibited that it can accurately perform image‐to‐image translation across brain MRI contrasts. It could hold great promise for clinical use for improved clinical decision‐making and better diagnosis of brain cancer patients due to the availability of multicontrast MRIs. This approach may be clinically relevant and setting a significant step to efficiently fill a gap of absent MR sequences without additional scanning.

## 1 INTRODUCTION AND RELATED WORKS

### 1.1 Introduction

Magnetic resonance imaging (MRI) is recognized as the preferred imaging modality for soft tissues. It has widely been utilized in brain soft tissue imaging. Various MR intramodality image contrasts can be acquired by applying different imaging protocols. These sequences are associated with diverse visualization and noise mechanisms that capture unique characteristics of the underlying anatomy. Multicontrast MR images provide clinicians with comprehensive clinical information for better diagnosis and treatment (e.g., accurate delineation of brain structures on the MR images) of brain cancer patients. Each image contrast provides a unique view of intrinsic MR parameters. However, obtaining multicontrast images for each patient is not always achievable due to the unavailability of sophisticated scanners in many centers and adding extra cost to the patient. Consequently, these factors may contribute to the loss of critical information due to missing complementary image contrast.

Intramodality and intermodality image synthesis is an active area of research in radiation oncology and radiology fields. The traditional model‐based image synthesis methods require predefined rules and tuning of parameters for every case and cannot achieve satisfactory results.^1^ The rapid growth of deep learning and computer vision algorithms has inspired researchers to investigate the data‐driven methods for image synthesis (also known as “adaptive domain”) due to their superior mapping capability of nonlinear relationships.^1^ Deep learning‐based image synthesis methods are an emerging field of research and are more generalizable than conventional model‐based methods. In other words, the model of a given image‐to‐image translation modality can be generalized to different image modalities.

### 1.2 Related works

Convolutional neural network (CNN) and its variant architectures such as part‐based CNNs,^2^ deep salient object detectors without expert annotation,^3^ Eliminating Indefiniteness Net,^4^ U‐Net,^5^ and GANs^6^ can be implemented for MR image synthesis with improved accuracy. MR image synthesis task could be approached either through image‐to‐image (single‐input and single‐output) or multistream (multi‐input and single‐output) translations. Image‐to‐image translation approach (e.g., model receives a single source contrast and learns the latent representation sensitive to distinctive features of the source) was studied by several investigators across MR sequences using dilated CNN,^7^ U‐Net,^8^, ^9^, ^10^ and variant generative adversarial networks (GANs) including edge‐aware GAN,^11^, ^12^ conditional‐GAN,^10^, ^13^, ^14^, ^15^, ^16^ diamond‐GAN,^17^ cycle GAN,^18^, ^19^ and unified‐GAN.^20^, ^21^ On the other hand, the multistream translation approach (e.g., model receives multiple unique sources and learns the shared latent representation more sensitive to general features across sources) to generate a missing or corrupted image contrast was studied using different CNN architectures.^7^, ^15^, ^22^, ^23^, ^24^, ^25^, ^26^ These proposed models for cross‐sequence MR image synthesis have revealed promising results for various applications in radiotherapy and radiology including, improving the brain tumor and healthy critical organs segmentation,^19^, ^27^ tumor characterization in neuro‐oncology,^10^, ^22^ and generating MR angiography images.^9^

Even though cross‐sequence MR synthesis was widely studied either using a single image or multiple images as input, a few of these studies provide comprehensive experimental results on a unified dataset.^7^, ^21^ Only one study^21^ utilized a convolutional deep learning model for image‐to‐image translation across multiple standard MRI contrasts for the brain using a relatively large‐scale open‐source dataset from multi‐institution. Dai et al.^21^ studied a unified‐GAN model for image‐to‐image translation across four MRI sequences. Despite the promising reported results, there is still a need for exploring other methods with improved generalizability for image‐to‐image translation across different MR sequences.

U‐Net architecture, a class of deep learning algorithms that belongs to the fully CNNs, has been used as a standard for semantic image segmentation where it showed outstanding performance.^5^ Its training procedure is efficient when using limited computational resources compared with GANs. Moreover, it can derive local and global features from the input images to generate pixel/voxel‐wise predictions. Inspired by its success in the image segmentation task, we studied an end‐to‐end deep learning convolutional U‐Net model for image‐to‐image translations across three standards MRI contrasts for the brain using a relatively large‐scale open‐source dataset from multiple institutions. Among the existing MR contrasts, each image contrast can be translated to the rest contrasts using the appropriate model. This is clinically helpful in situations where multiple MRI contrasts are needed for diagnosis and treatment planning, but only one MRI contrast could be practically acquired. We also compared our results with those achieved with state‐of‐the‐art models in the literature that utilized the same dataset. Our end‐to‐end 2D U‐Net model proposed in this study for image‐to‐image translation is developed by typically performing the following modifications on the original 2D U‐Net architecture proposed by Ronneberger et al.^5^: (a) replacing the sigmoid activation function in the final layer with tanh as we are treating the synthesis as a regression task, (b) adding dropout layers to prevent model overfitting, (c) implementing zero‐padding before every convolution operation to maintain the image size constant, and (d) substituting the up‐convolutional layers with up‐sampling. Despite its simplicity, our 2D U‐Net model provided accurate predictions by generating synthetic images with encouraging quality. The model can be used to produce a resembled 3D synthetic‐MR image slice‐by‐slice. As the computational expense grows with extending the proposed 2D U‐Net network in this study to full 3D that requires more GPU memory,^28^ we will consider this in the future to account for adjacent information between MR slices for better prediction quality.

## 2 MATERIALS AND METHODS

### 2.1 Imaging dataset

The imaging dataset used in this study was obtained from the BRATS’2018 Challenge for Brain Tumor Segmentation.^29^, ^30^, ^31^, ^32^, ^33^ The BRATS dataset consists of four multicontrast MRI scans of 477 patients with high‐grade or lower‐grade glioma brain cancer. The multicontrast MRI scans/subjects include T1‐weighted gradient‐echo (T1), T1‐weighted post‐gadolinium contrast gradient‐echo (T1‐Gd), T2‐weighted gradient‐echo (T2), and T2‐weighted fluid‐attenuated inversion recovery (T2‐FLAIR) or simply “FLAIR” provided for each patient. The datasets were collected from several institutions. The images were acquired using two types of scanners, GE and Siemens. Besides, the scans were obtained using different magnetic fields (1.5 and 3 Tesla) and various imaging protocols. All scans were acquired in axial plane/acquisition with slice thicknesses ranging from 1 to 5 mm. The typical MRI subject/scan comprises 155 slices taken across the axial plane, with each slice having a dimension of 240 × 240 pixels and a voxel size of 1 mm^3^. The entire data were randomly divided into 80% as the development set and the remaining 20% as the test set; the development set was further split into 80% assigned as the training set and 20% as the validation set. As we compare the results of multiple image‐to‐image translation algorithms trained on the same dataset pool, we used a random seed to produce a predictable sequence of numbers to ensure that all models use the same validation and testing data sets for a fair evaluation.

### 2.2 Preprocessing

Data preprocessing is an essential step in any deep learning framework, where the data are processed before feeding into the network. BRATS’2018 data were originally provided with initial preprocessing. Within the patient subjects, the data of each MR contrast (T1, T2, and FLAIR) were rigidly coregistered slice‐by‐slice to the T1‐Gd contrast. Then, they were resampled to 1 mm^3^ resolution and skull stripped. With regard to image registration for the brain region, precise aligning of the structures such as white and gray matters is critical for monitoring brain disease.

We applied further preprocessing to the MRI data, including null slices removal, image resizing, and intensity normalization and scaling. *First*, we performed dimensional reduction on the MRI subjects to remove null slices. Each MRI subject in the original BRATS’2018 dataset had 155 slices with a dimension of 240 × 240 pixels. However, not all those slices contain anatomical information and are useful for network training. As a result, we excluded those null slices and retained only ones that contain brain tissue, yielding about 100 slices per subject. *Second*, we applied an MRI intensity standardization approach to reducing the adverse impact of signal variation across different scanners and using various clinical acquisition protocols. Following the *z*‐score (zero mean and unit variance) standardization method, we computed the mean and the standard deviation (SD) of the intensities over the brain volume. We normalized the MR subject to having zero mean and unit SD; we then shifted the normalized data across each subject to have the pixel values within a [0, 1] scale. It has been reported that applying intensity normalization before training the model improves the MR image synthesis results.^34^ Applying a normalization method would prevent bias in quantitative assessments, ensuring optimal model training and comparable intensity ranges across subjects. *And third*, we resized the MR images to match the U‐Net network 2D input images. The provided MR image slices were resized to 224 × 224 pixel size by using cubic interpolation. All these preprocessing procedures were applied to the source and target MR subjects of all contrasts before feeding them to the network for training. We used Python (version 3.7; Python Software Foundation, Wilmington, DE, USA) to perform this preprocessing.

### 2.3 Network architecture

The U‐Net architecture^5^ is a popular CNN structure that was originally introduced for biomedical image segmentation. It has a symmetric hierarchical structure that composes an encoder and a decoder part with skip connections utilized for pixel‐wise prediction. The encoder (contraction path) down‐samples the input images to extract larger sets of low‐ to high‐level features. On the other hand, the decoder (expansion path) takes the output of the encoder and combines extracted image features in multiscale resolution levels to generate targeted image output through an up‐sampling process. Skip connections are added between mirrored layers in the encoder‐decoder network to speed up information transmission between input and output image flows. They help to learn matching features for corresponding mirrored layers.

**FIGURE 1:**
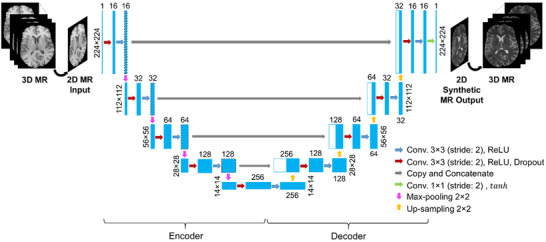
Our U‐Net architecture for MR image‐to‐image translations. Each blue box represents a set of feature maps. The number on top of the box donates the extracted feature maps, and that at the left/right side of the box represents the size of feature maps. White boxes represent copied feature maps. The arrows denote the different operations

The U‐Net configuration implemented in this study to perform cross‐sequence MR image‐to‐image translation (Figure 1) was adapted from the standard architecture.^5^ In total, our proposed U‐Net architecture consists of 19 convolutional layers. The input images have a size of 224 × 224 pixels and one channel (grayscale image). The encoder consists of a repeated implementation of two 3 × 3 convolutions with 2 pixels stride over five layer‐blocks, except the last bock that is made up of one convolutional layer. Zero padding was used before convolution to maintain the resolution of extracted deeper feature maps matching the resolution of the input feature maps. The first convolutional layer is followed by a rectified linear unit (ReLU) activation^35^ layer and a dropout operation,^36^ whereas the second convolutional layer is followed by a ReLU activation layer and a 2 × 2 max‐pooling operation with a stride of 2 pixels. Using a ReLU nonlinear transfer function between the hidden convolutional layers has the advantage of computational simplicity and representational sparsity providing capabilities for better solutions (it does not suffer from the vanishing gradient issues). Dropout regularization was implemented to reduce the likelihood of model overfitting with an increasing rate from 10 to 30% across the multiscale resolution levels in the network. Applying a max‐pooling operation after the activation layer reduces the spatial size of the image feature map by a factor of 2 which decreases the computational cost and saves memory. The number of convolutional filters (extracted feature maps) increases by a double from 16 in the first block to 256 in the last one. This permits the network to learn the hierarchical relationships over a large receptive field of the MR image. Because we do not have a GPU memory leverage at our computational architecture, we used this relatively smaller setting than that in the standard U‐Net (64 to 1024).

The decoder part is typically a mirror version of the encoder network. The main exception is that the max‐pooling operations in the encoder part were replaced with un‐pooling/up‐sampling operations in the decoding part. The up‐sampling process in the decoder part uses a nearest‐neighbor interpolation, which increases image size by a factor of 2 through each layer. Up‐sampling is used instead of deconvolution because the latter suffers from checkerboard artifacts caused by random initialization problems and an unequaled kernel size of the deconvolution concerning the stride. The encoder and the decoder parts are connected through skip connections at multiscale resolution levels to help recover the original spatial resolution of the input image at the output. The features from each block in the encoder were copied and concatenated with their corresponding ones in the decoder. These concatenations enable both high‐ and low‐level features from the encoding part to be utilized as additional inputs in the decoding part to provide effective and stable image representation. The output layer of our U‐Net composes of a 1 × 1 convolutional layer and a stride of 1 followed by a hyperbolic tangent (tanh) activation function. Among the examined activations, for example, linear, ReLU, and tanh for image synthesis task in this study, tanh was found to provide the best result. The final layer reconstructs an output image from a 16‐component vector of feature maps that has the same size as the input image (224 × 224).

### 2.4 Training the network

The U‐Net model proposed in this study implements 2D convolutions and only supports 2D input images. After preprocessing the data, the total number of 2D images of all data sets was ∼47 700 (∼100 slices × 477 subjects) slices for every MRI contrast. We trained our U‐Net model from scratch for each image translation. The model trainable parameters (weights and biases) were initialized using He et al.^37^ uniform distribution technique. This initialization method has shown better performance than the Xavier technique for deep models with ReLU layers. Adam stochastic optimization algorithm^38^ with a learning rate of 0.001 is applied to minimize a mean‐squared error (MSE) loss function in a stepwise fashion and update the network's trainable parameters at every training step progressively until the model reaches the convergence. We chose the MSE as a cost function because it is computationally inexpensive and leads to a convex optimization problem with a stable gradient. Other loss functions such as mean absolute error (MAE) were also investigated; however, its performance was inferior to MSE. The batch size, defined as the number of samples per gradient update, was set to 32 slices to make the best use of our CPU memory and computational power while does not negatively affect the model performance. Generally, a batch size of 32 is a good choice and works fine, smaller batch size is found to hurt the performance (e.g., model robustness/stability or generalizability). The number of epochs to train the network was set to 120, resulting in a total of 4320 iterations. This number of epochs was enough to reach training loss convergence. At each iteration, a batch of 32 images was randomly chosen from the training dataset for training the networks.

Our end‐to‐end 2D U‐Net architecture for image‐to‐image translation resulted in a total of ∼2.0 million trainable parameters. These parameters were optimized during the model training on the training data set to learn mapping a source MR contrast to a target contrast. During the training, the fitness of the model was regularly verified on the validation data set. To prevent the possibility of the model overfitting problem, we applied an early‐stopping method in addition to the dropout regularization technique. The early‐stopping technique terminates the training process early when there is no improvement in the model performance. We monitored the validation loss by passing it to early‐stopping where we have set the patience parameter to 20. The patience parameter is the number of epochs to check for improvement. We repeated the training processes several times to ensure that the best solution was obtained and avoid the problem of the initial seed of the optimization procedure. The model that exhibited the best performance on the validation data set was selected. The training was executed on a 64‐bit Windows Operating System, with an Intel Core i5 CPU and 8 GB RAM that took around 34 h. Once the model's training is finalized, it can be used for image‐to‐image translation on the test set to generate a resembled 224 × 224 × 155 volumetric image slice‐by‐slice in <1 min. Individual 2D U‐Net models were trained to perform the possible translations across the T1, T2, and FLAIR contrasts as follows: T1→T2, T2→T1, T1→FLAIR, FLAIR→T1, T2→FLAIR, and FLAIR→T2. The training of all models was performed using Keras API (version 2.6) with Tensorflow (version 2.6) as the backend in Python (version 3.7; Python Software Foundation).

### 2.5 Model evaluation

The quality of the generated MR images with U‐Net was evaluated against the ground‐truth images using four pixel‐wise metrics: MAE, MSE, peak signal‐to‐noise ratio (PSNR), and structural similarity index (SSIM) metric.^39^ These metrics take into account the quantitative as well as qualitative differences that mimic human perception. The formula of MAE is given as: $\mathrm{MAE}\;\left(x,y\right)=\frac{1}{n}\;\sum_{i\;=\;1}^{n}\left|y_{i}-x_{i}\right|$, where n is the total number of pixels or data points, $x_{i}$ and $y_{i}$ are the ground‐truth and predicted values, respectively. MSE metric is strongly dependent on the scale of intensities; therefore, we followed similar intensity normalization procedures for fair comparison. It is mathematically defined as: $\mathrm{MSE}\;\left(x,y\right)=\frac{1}{n}\;\sum_{i\;=\;1}^{n}{\left(y_{i}-x_{i}\right)}^{2}$. PSNR measures if the synthesized MR is an evenly or sparsely distributed prediction. This metric takes into account both the MSE and the largest possible intensity value of the image. The PSNR is defined as: $\mathrm{PSNR}\;\left(x,y\right)=\;10\;\times \mathrm{lo}g_{10}\left(I_{\max}^{2}/\mathrm{MSE}\right),$ where $I_{\max}^{2}$ is the maximum pixel value of the image that depends on the datatype. SSIM attempts to capture the human perceived quality of images by comparing two images. Its formula is given as: $\mathrm{SSIM}\left(x,y\right)\;=\frac{\left(2\mu_{x}\mu_{y}+C_{1}\right)\left(2\sigma_{xy}+C_{2}\right)}{\left(\mu_{x}^{2}+\mu_{y}^{2}+C_{1}\right)\left(\sigma_{x}^{2}+\sigma_{y}^{2}+C_{2}\right)}\;,$ where μ is the mean image intensity, $\sigma^{2}$ is the variance of the image, $\sigma_{xy}$ is the covariance of the ground‐truth (x) and predicted (y) images, and $C_{1}$ and $C_{2}$ are constants added to stabilize the division with a weak denominator. PSNR and SSIM serve as widely used measures to assess overall quality, primarily capturing features dominated by lower spatial frequencies. To visually inspect the difference between the synthetic and real MR images, difference maps or residuals were computed as: $\left(x_{i},y_{i}\right)=\;y_{i}-x_{i}$. The evaluation was performed on the normalized images.

## 3 RESULTS

### 3.1 Image quality

**FIGURE 2:**
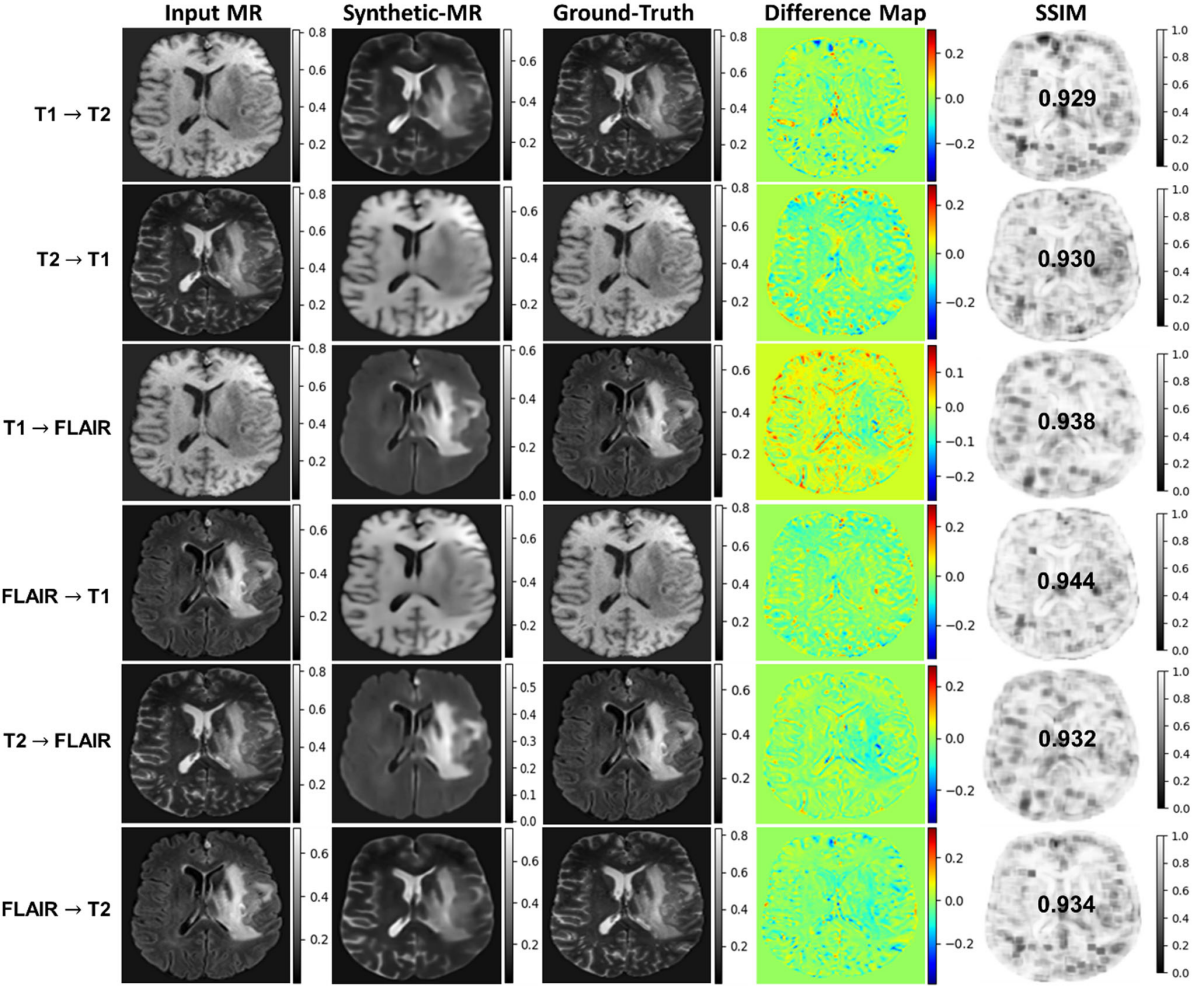
The comparison of synthetic‐MR images generated using a U‐Net model for one subject on a test data set. From left to right: input MR images (source image contrast); synthetic‐MR images (target image contrast); ground‐truth/real MR image (target image contrast); difference map (predicted–real MR image); and SSIM map. Rows show image‐to‐image translations across T1, T2, and FLAIR MR contrasts

The results of the synthetic‐MR images generated using a U‐Net model are presented in this section for all translations. These cross‐sequence translations involve synthesizing T1 from T2, T1 from FLAIR, T2 from FLAIR, and vice versa. The synthetic‐MR images compared with their corresponding ground‐truth ones are shown in Figure 2 for one example patient on the testing dataset. Visually inspecting the synthetic images shows that they are nearly indistinguishable from their corresponding real ones. As shown in the figure, our model can effectively synthesize tumor regions. Synthetic FLAIR images exhibited lower visual quality compared to other translated images, represented in a noticeable loss of details in the brain tissue on the image. The difference map, intensity difference between the synthetic and ground‐truth images, shows small residuals of image intensities. Most of the anatomical structures were preserved in the synthesized images. The SSIM metric, which measures the degree of similarity to ground‐truth MRI images, has indicated high similarity between the ground‐truth and synthesized images with values above 0.93.

The quantitative results of synthetic‐MR images compared with the ground‐truth images are presented in Table 1 for each translation on the test data set using the MSE, MAE, SSIM, and PSNR evaluation metrics. The lower MAE/MSE and higher PSNR/SSIM values (shown in Table 1 with arrows) exhibit better results. The average value of the SSIM was above 0.93 for all translations, indicating how the synthetic images were structurally similar to the ground‐truth images.

**TABLE 1:** Quantitative results of synthetic‐MR images using U‐Net compared with the ground‐truth images evaluated using PSNR, MSE, MAE, and SSIM metrics on the test dataset for all translations

Translation	PSNR ↑ (dB)	MAE ↓	MSE ↓	SSIM ↑
T1→T2	29.45 ± 1.72	0.0124 ± 0.0027	0.0012 ± 0.0004	0.932 ± 0.023
T2→T1	29.44 ± 1.85	0.0149 ± 0.0050	0.0012 ± 0.0005	0.937 ± 0.020
T1→FLAIR	33.25 ± 1.55	0.0086 ± 0.0020	0.0005 ± 0.0002	0.946 ± 0.013
FLAIR→T1	30.73 ± 1.81	0.0125 ± 0.0038	0.0009 ± 0.0004	0.946 ± 0.017
T2→FLAIR	33.01 ± 1.66	0.0089 ± 0.0027	0.0005 ± 0.0002	0.944 ± 0.015
FLAIR→T2	29.57 ± 1.78	0.0120 ± 0.0036	0.0012 ± 0.0005	0.936 ± 0.022

The vertical arrow's direction indicates the trend for better results and higher image qualities. Horizontal arrows indicate the direction of synthesis. Results reported as the mean ± 1 SD.

### 3.2 Model performance

**FIGURE 3:**
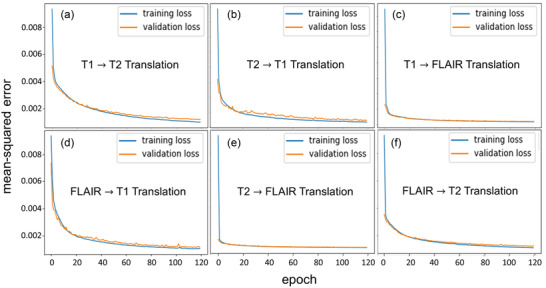
A plot of the training loss versus validation loss over the number of epochs for all translations. (a) T1 to T2, (b) T2 to T1, (c) T1 to FLAIR, (d) FLAIR to T1, (e) T2 to FLAIR, and (f) FLAIR to T2. The training learning curve shows the goodness of the model's learning, whereas the validation learning curve shows the goodness of the model's generalization

Visualizing the training loss versus validation loss over the number of epochs is an effective way to determine whether the developed model has been sufficiently trained. The learning curve of model performance on the training and validation datasets is widely used as a tool to diagnose the learning and generalization behavior of models (e.g., model exhibits underfitting, overfitting, or just well‐fitting performance). Underfitting happens when the model cannot achieve a satisfactorily low error value on the training set, whereas overfitting happens when the model fits the training dataset too well including the statistical noise or random fluctuations in the dataset. A just well‐fitting is recognized by a training and validation loss that decreases to a point of stability with a minimal gap between the two final loss values. Figure 3 shows the learning curves of our U‐Net model for the six translations. From the figure, we can notice that the U‐Net has comparable performance on both training and validation data sets. The learning curves demonstrated a continuous decreasing trend until reaching the stabilization (convergence) with the selected 120 epochs. The training loss is always lower than the validation loss and the gap between them is minimal (both curves are very similar), demonstrating good generalizability of our model for all translations. This also reveals that all models were well‐fit.

### 3.3 Comparison with other methods

To make the comparison meaningful, we compared our U‐Net results with all other models that used the BRATS dataset to develop their state‐of‐the‐art deep learning models for image‐to‐image MR translation. Comparison of the quantitative MAE, PSNR, and SSIM metrics indicated that our results were overall as good as the best‐reported results in the literature. We achieved the best MAE in all translations over the compared methods, which demonstrates the advantage of our model. Moreover, our PSNR and SSIM results were very similar to the best‐reported results with a maximum difference of 1.0 dB and 3.5% (0.035), respectively.

## 4 DISCUSSION

Taking a transition from a given MR image contrast (source) to another image contrast (target) can be performed through a cross‐sequence translation framework. Deep learning‐based methods are capable to learn this nonlinear mapping. In this study, we developed a deep convolutional U‐Net model to perform image‐to‐image translation across T1, T2, and FLAIR MRI contrasts for the brain.

The BRATS’2018 MRI datasets used in this study were obtained from different institutions (acquired on 1.5/3T MRI scanners and various acquisition protocols) for patients with brain lesions. The diversity in the collected datasets helps in developing a model that is robust to scanner types and setup practices as well as has a higher degree of generalizability. It has been known that the performance of a deep learning model depends on the amount of training data. Thus, the size of the entire dataset used in this study (∼47 700 images) was adequate for training our model for improved prediction accuracy. All MRI subjects used in this study were included image slices with pathologies (brain tumors/lesions) and healthy tissue structures, almost equally present in the dataset. Training a model on datasets that contain a mixture of images with normal and abnormal tissue structures enables producing a more robust model for real‐world applications. The provided BRATS data were further preprocessed before feeding the images to the network by removing null slices, resizing the images, and applying intensity normalization and scaling. The zero‐mean/unit‐variance intensity normalization technique that was applied in this study is less sensitive to high or low‐intensity outliers compared with others such as minimum/maximum normalization procedures. It also preserves the distribution of intensity values.

The proposed U‐Net model architecture (Figure 1) represents a complex mapping function that performs image‐to‐image translation across MR contrasts. The network architecture, optimization algorithm, hyper‐parameter selection, and the number of trainable parameters affect the model performance. As a result, we examined various parameter settings in developing our U‐Net model to determine the optimal possible performance in the image‐to‐image translation task. We assessed the impact of the inclusion of batch normalization in our U‐Net architecture on the prediction results. Based on trial‐and‐errors testing, we found that batch normalization does not affect the predicted results as we normalized that input data before feeding it to the network. Therefore, it was not included in our model. Applying intensity normalization to the input data helps in reducing the internal covariate shift of the training datasets for improved robustness and faster convergence. We also assessed replacing the up‐sampling operation in our U‐Net with transposed convolutions (filter size of 2 × 2 and stride = 2). The results showed that both operations provide almost similar results (PSNR and SSIM values); as a result, we kept the up‐sampling since it offers fast computation. The dropout layer in our network helps to reduce the risk of model overfitting that results in poor generalization of the model. Also, implementing a validation process on a hold‐out data set helps prevent the overfitting problem and improve the network's generalizability on unseen data during the training. The choice of the Adam optimization algorithm is because it offers faster convergence compared with the standard stochastic gradient descent methods.

The experimental results (Figure 2) demonstrate the capability of our model for accurately performing image‐to‐image translation on the test set. Side‐by‐side visual comparisons of the images revealed that the synthetic‐MR images were very similar to the real ones for all translations. Tumor regions were successfully synthesized by our model. Synthetic FLAIR MR images exhibited loss of details, blurring, and other imperfections. They had slightly lower quality compared with synthetic T1 and T2 MR images. This issue may highlight that there are limitations associated with generating this image contrast. The difference maps show small residuals; however, further improvement is required to have clinical utility. We can observe that there is a slight underestimation of the intensities as a trend across all translations. The performance learning curves (Figure 3) demonstrate a good fit of our models for image‐to‐image translation across different MR contrasts. In addition, the models show high generalizability, robustness, and stability. Compared with other state‐of‐the‐art deep learning models using BRATS datasets (Table 2), the results achieved by our model were comparable to the best results for MR image‐to‐image translations. Our MAE results were superior to all reported results achieved by other models^7^, ^11^, ^13^, ^14^, ^21^ for all translations. For the PSNR metric, our results were as good as the best results reported for T1→T2 translation by Chartsias et al.^7^ (29.45 vs. 30.96 dB), T2→T1 by Dai et al.^21^ (29.44 vs. 30.16 dB), T1→FLAIR by Chartsias et al.^7^ (33.25 vs. 30.32 dB), FLAIR→T1 by Dai et al.^21^ (30.73 vs. 30.16 dB), T2→FLAIR by Dai et al.^21^ (33.01 vs. 28.98 dB), and FLAIR→T2 by Dai et al.^21^ (29.57 vs. 30.11 dB). Similarly for SSIM metric, our results were comparable to the best results that were reported by Dai et al.^21^ (0.932 vs. 0.969) for T1→T2, Dai et al.^21^ (0.937 vs. 0.950) for T2→T1, Yu et al.^11^ (0.946 vs. 0.963) for T1→FLAIR, Dai et al.^21^ (0.946 vs. 0.952) for FLAIR→T1, Dai et al.^21^ (0.944 vs. 0.958) for T2→FLAIR, and Dai et al.^21^ (0.936 vs. 0.970) for FLAIR→T2. The quality of the synthetic images produced by our model shows the potential for clinical use and could be beneficial in reducing the cost to the patient and shortening the acquisition time by reducing the number of scans required.

**TABLE 2:** Comparison of our U‐Net model performance on the test data set with other state‐of‐the‐art deep learning models trained on the BRATS dataset

Translation	Study (year)	Network	PSNR ↑(dB)	MAE ↓	SSIM ↑
T1→T2	Yang et al.^14^	Conditional‐GAN	22.56	8.292	0.866
	Dai et al.^21^	Conditional‐GAN	30.02	0.041	**0.969**
	Yu et al.^11^	Edge‐aware‐GAN	29.98	0.088	0.967
	Dar et al.^13^	Conditional‐GAN	27.19	——	0.946
	Chartsias et al.^7^	Dilated‐CNN	**30.96**	0.333	0.929
	This study	U‐Net	29.45	**0.012**	0.932
T2→T1	Yang et al.^14^	Conditional‐GAN	22.52	9.937	0.854
	Dai et al.^21^	Conditional‐GAN	**30.16**	0.058	**0.950**
	Dar et al.^13^	Conditional‐GAN	25.80	——	0.940
	This study	U‐Net	29.44	**0.015**	0.937
T1→FLAIR	Yang et al.^14^	Conditional‐GAN	22.69	7.934	0.837
	Dai et al.^21^	Conditional‐GAN	29.09	0.041	0.959
	Yu et al.^11^	Edge‐aware‐GAN	30.11	0.105	**0.963**
	Chartsias et al.^7^	Dilated‐CNN	30.32	0.283	0.897
	This study	U‐Net	**33.25**	**0.009**	0.946
FLAIR→T1	Dai et al.^21^	Conditional‐GAN	30.16	0.057	**0.952**
	This study	U‐Net	**30.73**	**0.012**	0.946
T2→FLAIR	Dai et al.^21^	Conditional‐GAN	28.98	0.042	**0.958**
	Yang et al.^14^	Conditional‐GAN	21.66	8.858	0.836
	This study	U‐Net	**33.01**	**0.009**	0.944
FLAIR→T2	Dai et al.^21^	Conditional‐GAN	**30.11**	0.040	**0.970**
	This study	U‐Net	29.57	**0.012**	0.936

**Bold** indicates the best results. The vertical arrow's direction indicates the trend for better results and higher image qualities. Horizontal arrows indicate the direction of synthesis.

While the proposed method in this study and Dai et al.^21^ implemented an image‐to‐image translation approach for comprehensive evaluation across multiple MR sequences, there is also another alternative approach based on multimodel image synthesis^7^ where more than one sequence are used as inputs to synthesize a single output sequence. Each strategy has its inherent weaknesses and strengths. For instance, the image‐to‐image translation strategy requires only one image sequence as input to synthesize multiple other complementary sequences. However, the quality of the synthesized images is relatively lower compared with the multimodal methods. On the other hand, multimodal strategy provides superior synthetic image sequence quality as they could capture more feature information for multiple distinctive sequences but gathering multiple MR sequences are not always possible. The multimodal strategy may be a useful complement to the image‐to‐image translation strategy. Regarding the current worldwide COVID‐19 pandemic, MRI synthesis can be utilized for eliminating the indefiniteness of the clinical spectrum for better screening. Different MR imaging modalities can be used for understanding the common features of COVID‐19 pneumonia for radiologists.^40^, ^41^ In this direction, this is particularly crucial for patients who require dynamic observation and for the examination of children and young people due to the ionizing radiation‐free nature of MR images.^41^ MR image synthesis could also be applied to improve the brain tumor structures contouring in radiotherapy treatment planning by generating the missing MRI sequence information.^27^ Radiotherapy treatment of brain cancer patients often requires more than one MR sequence for delineating the structures. Studies^19^, ^27^ have reported that brain lesions could be delineated on the synthesized MR images with reasonable accuracy; dice similarity coefficient between the synthesized and real brain MR images ranged from 0.71 to 0.97 for different sequences.

The developed model in this study only requires one MR image contrast to perform different translations with promising accuracy. Most importantly, our model uses standard MR image contrasts (T1, T2, and FLAIR) that are the most common general diagnostic MR sequences and do not need special pulse sequences. There are a few limitations that can be highlighted in this study as well. *First*, our model was developed using a 2D deep learning convolutional method for MR image synthesis due to computer memory and computational power restrictions. Future development may consider extending this work to a 3D approach to predict more accurate interslice information. *Second*, U‐Net performs pixel‐wise image synthesis based on paired data, requiring perfect image coregistration for proper model training. Thus, improper aligning of the paired MR images may cause losing some information of brain structures in the produced images.

## 5 CONCLUSION

We proposed a deep learning convolutional U‐Net to perform image‐to‐image translation across MRI contrasts for the brain. Our model exhibited competitive performance to the best state‐of‐the‐art developed models for this task using the BRATS data; however, we should highlight that the comparison with the state‐of‐the‐art methods was not performed based on the same test set. It generates synthetic‐MR images of remarkable visual similarity to the ground‐truth images on the testing dataset. Our results could hold great promise for clinical use for improved clinical decision‐making and better diagnosis of brain cancer patients due to the availability of multicontrast MRIs. This approach may be clinically relevant and setting a significant step to efficiently fill a gap of absent MR sequences without additional scanning.

## CONFLICT OF INTEREST

The authors have no conflict of interest to disclose.

## AUTHOR CONTRIBUTION

A. O. contributed to the conception and design of the study, developing the models, and drafting and revising the manuscript for important intellectual contents. N. T. contributed to writing and revising the manuscript. All authors contributed to manuscript revision and approved the submitted version.

## Data Availability

The datasets generated and/or analyzed during the current study are available in the BRATS 2018 challenge repository at https://www.med.upenn.edu/sbia/brats2018/data.html. The code for the U‐Net model developed in this study is also available as an open‐source package on GitHub respiratory at https://github.com/afiosman/UNet‐for‐MR‐to‐MR‐image‐translation.
